# What are forests for? Social perceptions of the functions of public-managed forests following mega-fire events

**DOI:** 10.1007/s13280-025-02200-1

**Published:** 2025-06-03

**Authors:** Elisabete Figueiredo, Eduarda Fernandes, Cristina Ribeiro, Carla Ferreira

**Affiliations:** 1 Department of Social, Political and Territorial Sciences and GOVCOPP – Research Unit on Governance, Competitiveness and Public Policies, University of Aveiro, Campus de Santiago, 3810-193 Aveiro, Portugal; 2 School of Technology and Management and CARME - Centre of Applied Research in Management and Economics, Polytechnic Institute of Leiria, Morro do Lena – Alto do Vieiro, Apartado 4163, 2411-901 Leiria, Portugal; 3 CoLab Forestwise, Quinta de Prados, Campus da UTAD, 5001-801 Vila Real, Portugal; 4 CETRAD – Centre for Transdisciplinary Development Studies, University of Alto Douro and Trás-os-Montes, Quinta de Prados, Pole II, ECHS, Room 1.14, 5000-801 Vila Real, Portugal

**Keywords:** Forest functions, Forest values, Mega-fire events, Public-managed forests, Social perceptions

## Abstract

Despite the growing research interest regarding the perceptions of forest-related stakeholders about forest functions and values, the views of social actors not directly involved in forestry have been largely overlooked, particularly in Southern European countries like Portugal. This article addresses this gap by analyzing the social perceptions regarding the functions of the *Matas do Litoral*, public-managed forests in the Centre Region of Portugal, severely impacted by the mega-fires of October 2017. Through a hierarchical cluster analysis of survey results collected from 1000 inhabitants of the adjacent parishes of the *Matas*, three groups were identified—“The Moderately Interested,” “The Comprehensively Interested” and “The Environmentally Interested”—based on significant differences regarding their perceptions of forest functions, the future of these areas, essential factors for effective forest management, the mega-fires and their consequences. Sociodemographic characteristics—particularly age and literacy levels—forest ownership, knowledge and familiarity with these forests help to explain the differences between the three clusters.

**Supplementary Information:**

The online version contains supplementary material available at 10.1007/s13280-025-02200-1.

## Introduction

### The context

Forests are essential for communities and nations, providing a range of direct and indirect economic, environmental, and sociocultural functions and services and corresponding benefits, contributing to people’s economic and social well-being (Valente et al. 2015; Frick et al. 2018; Pour et al. 2023; Valente et al. 2024; Lehto et al. 2025). Over the last few decades, there has been a shift regarding the perceptions and demands of forest functions and services by society at large (Rametsteiner and Kraxner 2003; Rametsteiner et al. 2009; Dobsinska and Sarvasova 2016; Valente et al. 2024). The dissemination of environmental values and nature-oriented leisure interests and activities (Rametsteiner et al. 2009) have played an important role in how forest-related stakeholders and the general public view forests’ current functions. Additionally, the increasing re-orientation within the European Union (EU) to bio-based societies and economies (Rametsteiner et al. 2009; Valente et al. 2015; Dobsinska and Sarvasova 2016) together with the increasing recognition of forests multifunctional character (Nijnik et al. 2010; Pastorella et al. 2016) contributed to the shift from mainly valuing the traditional forest and production-oriented functions to progressively prioritize environmental and social functions.

Besides their prominent roles and functions, forests may face various risks, particularly wildfires (e.g., Valente et al. 2015; Oliveira et al. 2020; Newman Thacker et al. 2025), deforestation and changing and competing land uses (Ranacher et al. 2017), abandonment (Pastorella et al. 2016), forest owners’ absenteeism (Valente et al. 2015), diseases, pests and invasive species (Rametsteiner et al. 2009). These threats may affect both forest uses and social perceptions regarding forest functions, as they have enormous impacts on forests’ conditions and the services they may provide (Frick et al. 2018).

Among the various threats associated with forests, wildfires are generally perceived as the major risk, especially by Southern European individuals (Rametsteiner and Kraxner 2003; Rametsteiner et al. 2009; Valente et al. 2015), due to their devastation potential (e.g., Duane et al. 2025). Furthermore, due to their increasing intensity, frequency and magnitude, wildfires represent a rising risk to many countries (Lidskog et al. 2019; Duane et al. 2025; Newman Thacker et al. 2025), particularly in Europe, where forests represent a prominent part of the landscape, covering around 44% (Ciesielski and Sterenczak 2018). Within Europe, Southern countries face additional risk, due to their particular context, weather conditions, the increasing likelihood of extreme weather events and of the frequency and recurrence of wildfires (e.g., Linley et al. 2022; Duane et al 2025).

According to Huidobro et al. (2024) and Duane et al. (2025), a range of terms is employed to characterize wildfires, including the term “mega-fires.” Nonetheless, this concept remains contested within the scientific literature (e.g., Duane et al. 2025), with several scholars (e.g., Stoof et al. 2024) critiquing it for its ambiguous and emotive nature—attributes often associated with its predominant use in mass media discourse. Despite this, the term mega-fires is increasingly adopted in the academic context to describe exceptionally large and severe wildfire events, particularly in response to their growing frequency, intensity, and societal and environmental impacts (Linley et al. 2022; Huidobro et al. 2024). Drawing on systematic literature reviews, both studies propose that mega-fires may be defined as extreme wildfire events characterized by their extensive size—typically exceeding 10 000 hectares—as well as their atypical behavior and far-reaching consequences. These fires often result from multiple, interconnected ignition sources. Therefore, the use of the term mega-fire(s) appears to be appropriate when referring to the Portuguese context, particularly given the scale and characteristics of the wildfires that have affected the country in recent years. For the purposes of consistency and terminological clarity, the term mega-fire(s) will be employed throughout this text.

Portugal is, in fact, one of the European countries most significantly affected by wildfires (e.g., Duane et al. 2025), which are seen as a major environmental threat, stemming from factors such as rural and agricultural abandonment, land-use changes, climate change, and ineffective forest management and fire prevention policies (Oliveira et al. 2020). Over recent years, not only has the frequency of forest fires increased, but their intensity and scale have reached unprecedented levels, destroying on average around 100 000 hectares of forest every year in a country where forests cover approximately 36% of the territory (Figueiredo et al. 2024a, b). The mega-fires of June and October 2017 resulted in the loss of 500 000 hectares, marking an unparalleled scale of destruction regarding the extent of the area burned and the impacts caused (ICNF 2023; Figueiredo et al. 2024a, b). Together, these events caused 116 fatalities and inflicted damages to thousands of homes, companies and infrastructure (Oliveira et al. 2020).

Between 14 and 16 October 2017, 523 forest fires occurred simultaneously in different municipalities of Portugal (CTI 2022), destroying around 240 000 hectares (ICNF 2023). Some of these fires severely affected the *Matas do Litoral*—public-managed coastal forests located in the Centre Region of Portugal, that are part of the 3% of public forests in the country and are managed by a State agency, the Institute for Nature and Forests Conservation (ICNF). 67% (24 512 hectares) of the area of the *Matas* was destroyed in consequence of these fires (Tomé 2018), which mobilized more than 1000 firefighters and other firefighting resources. The fires of October 2017 impacted hundreds of homes, companies, and public infrastructure, although no fatalities were registered in the *Matas area*. One of the oldest forests in Portugal—the National Pinewood of Leiria (*Mata Nacional* (MN) *de Leiria*)—with exceptional symbolic and material values at local, regional and national levels, was also severely affected. 80% (around 9000 hectares) of its area destroyed. Due to its vast natural patrimony established over more than 700 years this MN is for long recognized as the ‘greatest monument of the country’ (Pinto 1938). The October 2017 wildfires in the *Matas do Litoral* were fueled by extreme weather conditions (wind and dry), which were intensified by the Hurricane Ophelia, causing the rapid progression and multiplication of the events (CTI 2022). As referred to in the CTI report, these wildfires were unique within the European Union area and can be qualified as mega-fires due to their extended duration, unusual large dimension, extension of the area burned and the complex interaction between individual fires (CTI 2022).

### Aims and objectives

This article explores the contrasting social perceptions regarding forest functions, taking the *Matas do Litoral* as a case study. These areas play a prominent role in nature conservation, maintenance of forest ecosystems’ functions, protection of high-conservation value areas and provision of management-related public services (Figueiredo et al. 2024a, b). Over recent years, social perceptions of specific stakeholders, user groups, and forest owners regarding forest functions have increasingly become interesting topics of research. However, as noted by Rametsteiner and Kraxner (2003), Rametsteiner et al. (2009) and Ciesielski and Sterenczak (2018), the analysis of the views concerning forest values and functions is somehow less abundant, particularly regarding social actors not directly related to forests and public-managed areas. In Southern European countries (like Portugal), the lack of these types of information and analyses is even more evident.

This article aims to contribute to filling this gap by analyzing the (diverse) social significance of forests in public-managed areas following mega-fire events. Understanding how public forests are socially perceived and what local populations deem essential regarding forest functions, values, and services is especially relevant to further inform sustainable forest management decisions and strategies (e.g., Edwards et al. 2012; Fabra-Crespo et al. 2012; Paletto et al. 2013a, b; Valente et al. 2015; Frick et al. 2018). This study adds further reflection based on data collected from a survey conducted to a sample of residents (*N* = 1000) of the *Matas do Litoral* neighboring parishes. Through a hierarchical cluster analysis, this study unveils the differences regarding the perceptions of forest values and functions, the key factors considered essential for effective forest management, the importance attributed to the *Matas*, the mega-fires’ impacts experienced, and the visions regarding the future of these areas.

## Literature review

The transformations in the social perceptions and demands regarding forests values and functions align with the increasing recognition of forest multifunctionality, that is, the acknowledgement of the simultaneous and interrelated diverse functions that forests can provide (Nijnik et al. 2010; Pastorella et al. 2016). Multifunctionality is increasingly considered crucial, within forest management, for the provision of several goods and services to society (Pastorella et al. 2016). Within the international literature, these services have been conceptualized as ecosystem services that can be broadly defined as the benefits obtained from forest’s ecosystems (e.g., Paletto et al. 2013b; Frick et al. 2018; Pour et al. 2023; Lehto et al. 2025). Although various classifications of forest functions have been used according to different contexts and purposes (e.g., Carvalho-Ribeiro and Lovett. 2011; Paletto et al. 2013b), this article primarily adopts the classification—mainly based on UNCED (1992)—from Paletto et al. (2013b), which organizes forest services into three main functions: (i) economic values, related to the production of wood and non-wood products, and job creation and maintenance; (ii) environmental values, associated with the protection of water, soil, biodiversity, climate regulation and protection from natural hazards; (iii) and social values, linked to recreation and leisure, preservation and protection of cultural heritage and patrimony, promotion of local identities and connections with forests.

As, among other, Rametsteiner and Kraxner (2003), Paletto et al. (2013b), Valente et al. (2015) and Dobsinska and Sarvasova (2016) conclude, forests are increasingly understood as natural environments, reflecting the needs, interests, values and preferences of contemporary societies. As Paletto et al. (2013b) indicate this represents a shift from more instrumental values to intrinsic ones. Intrinsic values are related to non-anthropocentric views (including esthetic, cultural and spiritual ones) and supporting the conservation of the integrity of the forest ecosystems. Instrumental values are related to anthropocentric views, based on the satisfaction of human and social needs, often connected to forests’ economic functions (Paletto et al. 2013b). Both types of values may coexist and shape different forest perceptions and preferences.

 Concomitantly, this also reflects growing social and political concerns regarding threats to forests, environment and biodiversity, namely—but not exclusively—wildfires and deforestation that have been recognized by forest managers as important to improve forests’ sustainable management (Dobsinska and Sarvasova 2016).

Sustainable forest management has equally evolved from just focusing on forests’ productive roles to integrating a broader range of services and functions (Schmithüsen and Seeland 2006), aiming at balance sociocultural, economic and ecological needs of both the present and the future generations (Paletto et al. 2013a, b; Valente et al. 2015). This supposes the integration of multiple (and new) stakeholders in the decision-making and management processes, not only to understand their different interests and needs but also to increase social acceptance and reduce potential conflicts (Fabra-Crespo et al. 2012; Paletto et al. 2013a, b; Pastorella et al. 2016; Pour et al. 2023).

Perceptions mainly relate to how people process information coming from their surrounding environments (Barona et al. 2022) and may be influenced by several factors, from individual (e.g., tastes, views, emotional states, age, gender) to contextual and experiential ones. The latter are related to sociocultural factors (as history, traditions, shared norms and values) and socioeconomic conditions, as well as to the physical characteristics of the perceived environment. Perceptions play a fundamental part in explaining people’s different behaviors, needs, interests, attitudes and decisions regarding what surrounds them, including forests (Barona et al. 2022).

The analysis of forest-related social perceptions highlights the relevance of socioeconomic characteristics (e.g., gender, age, literacy levels) in explaining the differences among stakeholders (e.g., Rametsteiner and Kraxner 2003; Rametsteiner et al. 2009; Edwards et al. 2012; Paletto et al. 2013a, b; Ranacher et al. 2017; Frick et al. 2018; Barona et al. 2022).

Overall, these studies stress that women tend to value forest functions highly than men, especially those linked to esthetic and spiritual values, as well as to environmental aspects, namely the conservation of natural resources and patrimony for the future generations. In contrast, men tend to attribute higher relevance to economic and productive functions, as well as to recreational and leisure-related values. Younger people and individuals with higher literacy levels tend to assign higher importance to cultural, spiritual and environmental values, while older and less educated individuals typically assign higher relevance to social and economic roles.

In the few studies addressing social perceptions about forests in Southern European countries (Rametsteiner and Kraxner 2003; Rametsteiner et al. 2009; Carvalho-Ribeiro and Lovett 2011; Edwards et al. 2012; Valente et al. 2015, 2024), not many noteworthy differences were found compared to Central and Northern European nations. Exceptions are the slightly higher relevance attributed by Southern Europeans to the conservation of forests’ biodiversity and protection from negative natural hazards, especially wildfires. These threats also play an important role in shaping social perceptions regarding forests (Valente et al. 2015; Pastorella et al. 2016; Ranacher et al. 2017; Oliveira et al. 2020; Newman Thacker et al. 2025), as they significantly impact these areas’ conditions and functions, and the services forests can provide (e.g., Frick et al. 2018). Rametsteiner and Kraxner (2003), Schmithüsen and Seeland (2006) and Dobsinska and Sarvasova (2016) also conclude that forest owners and other stakeholders whose income depends on forest-related activities tend to value the economic and productive functions over the environmental and sociocultural ones (e.g., Valente et al. 2015).

The role of information and knowledge in shaping diverse social perceptions forests and their functions is also highlighted as important in a number of studies (e.g., Rametsteiner and Kraxner 2003; Pastorella et al. 2016; Ranacher et al. 2017; Ciesielski and Sterenczak 2018; Frick et al. 2018; Pour et al. 2023). More informed and knowledgeable people tend to attribute higher relevance to forests’ environmental and sociocultural functions over economic ones.

Although less studied, forests’ history, traditions and other sociocultural aspects are also considered important factors in shaping social perceptions (e.g., Schmithüsen and Seeland 2006; Paletto et al. 2013b; Ranacher et al. 2017). The connections between people and forests; the tradition of forestry in their living areas and landscape; the relevance of forests for local economies, cultures and identities contributes to a more positive appreciation of forests and their functions (Schmithüsen and Seeland 2006), often establishing forests as important symbols of the communities (e.g., Edwards et al 2012).

## Materials and methods

### Data collection

**Fig. 1:**
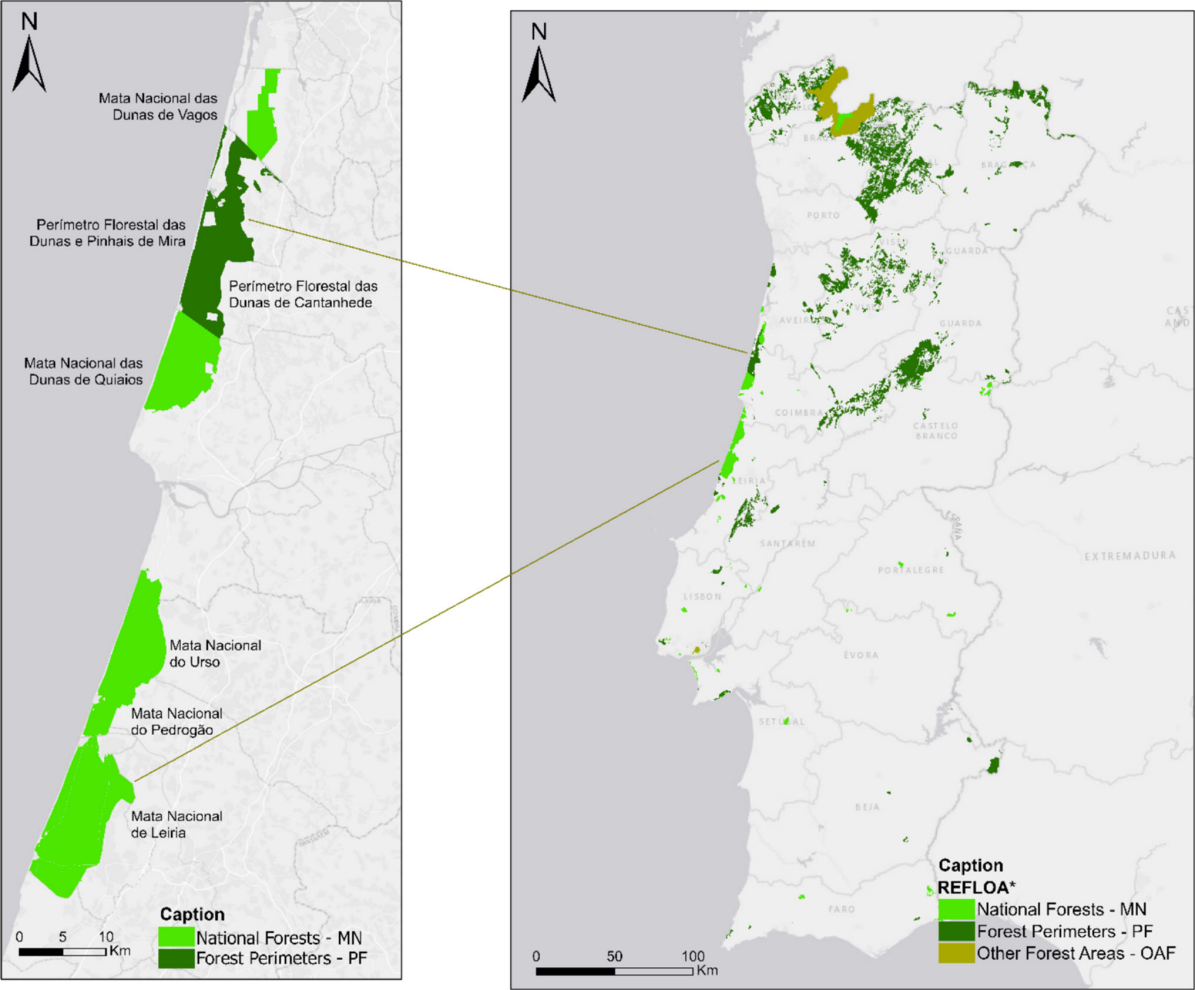
Map on the left: Location of the ‘Matas do Litoral’ analyzed. Map on the right: Location of the ‘Matas do Litoral’ within mainland Portugal. *Source*: Own elaboration, based on *REFLOA (Forest Regime and Other Areas, that represents the delimitation of land submitted to the Forest Regime in mainland Portugal) (Accessed on December, 3, 2024)

This article takes the *Matas do Litoral* as case study to assess the perceptions of the residents in the parishes bordering these forests. *Matas do Litoral* are public-managed forests, including some (as the MN of Leiria) with a strong symbolic and historical value at local, regional and national levels. The *Matas do Litoral* considered in this study (Fig. 1)^1^ include those significantly affected by the mega-fires of October 2017: the National Forests (*Matas Nacionais—*MN) of Leiria, Pedrógão, Urso, Dunes of Vagos, and Dunes of Quiaios and the Forest Perimeters (*Perímetros Florestais*—PF) of the Dunes of Cantanhede and Dunes and Pinewoods of Mira. Together, these areas occupy 36 124 hectares spanned across three NUTS III, 7 municipalities, and 16 parishes. Altogether these parishes have a population of around 50 000 individuals. The study focuses on how the inhabitants from these parishes perceive the functions and values of the *Matas*, following the mega-fire events. To this end, a survey was used for data collection, as this is the most adequate and feasible method when dealing with large populations (following, for instance, Rametsteiner and Kraxner 2003; Paletto et al. 2013a, b; Pastorella et al. 2016). The survey was designed to sample of 1000 residents over the age of 18 (around 2.5% of the total population) and selected using non-probability quota sampling procedures based on the following criteria: (i) number of inhabitants in each parish, (ii) gender, and (iii) age.

The survey was carried out, between November 2023 and February 2024, by a professional agency using the computer-assisted telephone interviewing method (CATI). The questionnaire was elaborated based on the literature on social perceptions about forests, their functions, values, uses, and management practices (e.g., Rametsteiner and Kraxner 2003; Paletto et al. 2013a, b; Pastorella et al. 2016; Ranacher et al. 2017; Frick et al. 2018). The questionnaire included 27 mainly closed-ended questions grouped into five sections: (i) questions about the sociodemographic characteristics of the respondents (e.g., gender, age, literacy level); (ii) questions about the respondents’ relationship with the *Matas do Litoral* (e.g., knowledge and perceptions of these areas, frequency habits and motivations); (iii) questions regarding the perceived values and functions of the *Matas do Litoral*—the main focus of this article—as well as questions about what respondents considered to be an effective forest management; (iv) questions about respondents’ perceptions of the wildfires of October 2017 and their impacts; and (v) questions on the respondents’ visions about the future of these areas*.*

### Data analysis

A hierarchical cluster analysis was performed employing the Ward’s method and the Squared Euclidean distance to identify homogeneous groups of respondents, using SPSS (Statistical Package for Social Sciences, version 29, IBM, USA). The hierarchical cluster analysis was based on the respondents ranking of 15 different forests’ functions and uses on a 5-point Likert scale (from 1—“not important” to 5—“extremely important”) in response to the question, “In your opinion, what are the most important functions and uses of the *Matas do Litoral*?”. The agglomeration schedules and dendrogram obtained suggested the existence of two, three or four clusters. To determine the optimal cluster solution, the approach outlined by Hair et al. (1998) was followed, profiling the clusters from the three solutions using the 15 variables that served as input for the analysis. A comparison of the results indicated that the three-cluster solution displayed the most distinctive profiles, being selected for further analysis. ANOVA and Chi-square tests were used to identify statistically significant differences among the three clusters regarding various dimensions: perceptions of *Matas do Litoral* prior to the fire events; knowledge, frequency, and motivations for visiting these areas; importance attributed to these forests; perceptions about the mega-fires of October 2017, of those responsible, impacts experienced and support received; perceptions about the most important aspects for the *Matas* effective management; interest and motivations for participating in management decision-making processes; perceptions of the future of *Matas do Litoral*; and the sociodemographic profiles of the respondents.

## Results

### Sample profile

The majority of the respondents are women (51.7%), married (60.6%) and aged between 25 and 64 years (61.7%). Most respondents are employed (55.5%), with a household net monthly income between €706 and €3000, and holding a university degree (43.9%). 21.4% of respondents are forest owners and, among these, 21.5% have properties bordering with at least one of the *Matas do Litoral* analyzed (Table S1).

Examining the importance that respondents attributed to the various uses and functions of the *Matas do Litoral* (Table 1), conservation and protection functions like “improving air quality” (4.76*),* “preserving nature and landscapes” (4.72), and “preserving diversity of animals and plants” (4.72) clearly stand out. On the contrary, economic functions such as the *“*production of non-wood products” (3.93) and “production of wood products” (3.71) are generally less valued. This aligns with the description of these areas (by 30.2% of the respondents) prior to the mega-fires of October 2017 (Table S2), through the use of words related to nature and the environment. However, 20.7% described these areas as abandoned, neglected, and lacking maintenance.

**Table 1:** Clusters’ profiles regarding the importance attributed to the functions and uses of the *Matas do Litoral* and of their overall relevance. *Measured using a Likert-type scale from 1—‘Not important’ to 5—‘Extremely important’. Values in bold correspond to the highest values when statistically significant differences exist

Importance of the Functions and Uses of the *Matas do Litoral*	Total sample (*N* = 1000)	Clusters			ANOVA	
	Mean	Cluster 1	Cluster 2	Cluster 3	*F* value	*p* value
		*The moderately interested* (*N* = 240, 24%)	*The comprehensively interested* (*N* = 350, 35%)	*The environmentally interested* (*N* = 410, 41%)		
		Mean	Mean	Mean		
Functions and uses of Matas do Litoral*						
Wood production	3.71	3.30	**4.95**	2.90	663.700	< 0.001
Non-wood products production	3.93	3.52	**4.97**	3.28	497.114	< 0.001
Production of biomass (energy)	4.12	3.66	**4.92**	3.70	290.067	< 0.001
Preservation of nature and landscapes	4.72	4.12	**4.96**	4.86	253.275	< 0.001
Provision of spaces for sports and recreation	4.48	3.66	**4.89**	4.61	308.363	< 0.001
Preservation of the historical patrimony	4.60	3.67	**4.95**	4.84	596.266	< 0.001
Preservation of the quality and availability of water	4.60	3.69	**4.95**	4.84	638.175	< 0.001
Protection and preservation of the soil	4.70	3.98	**4.94**	4.92	504.984	< 0.001
Creation and maintenance of jobs	4.18	3.48	**4.83**	4.05	236.566	< 0.001
Preservation of animals and plants diversity	4.72	4.00	**4.97**	4.92	480.422	< 0.001
Climate regulation	4.71	3.98	**4.97**	4.90	557.573	< 0.001
Improvement of air quality	4.76	4.10	**4.98**	4.97	416.457	< 0.001
Contribution to fight climate change	4.68	3.89	**4.95**	4.92	469.605	< 0.001
Preservation of cultural traditions	4.36	3.47	**4.91**	4.40	339.012	< 0.001
Promotion of environmental education and awareness	4.56	3.68	**4.91**	4.76	428.505	< 0.001
Importance of Matas do Litoral*						
To your parish	4.50	3.96	**4.86**	4.51	82.929	< 0.001
To your municipality	4.50	4.00	**4.84**	4.51	71.921	< 0.001
To the Centre Region of Portugal	4.66	4.08	**4.93**	4.75	164.507	< 0.001
To the country	4.76	4.26	**4.93**	4.90	160.498	< 0.001
Internationally	4.51	3.91	**4.83**	4.58	113.082	< 0.001

Although respondents ranked the importance of the *Matas do Litoral* (Table 1) higher than 4.5, their relevance is mainly recognized at the country (4.76) and the region (4.66) levels. Respondents particularly familiar with the MN of Leiria (74%) and MN of Pedrógão (60%), while knowledge of the other five *Matas* is notably lower. The MN of Leiria (51.1%) and Pedrógão (30.1%) are the most frequently visited, mainly to “admiring the landscape and contacting with nature” (35.1%) and “hiking or walking*”* (26%). For most respondents (54.3%), the mega-fires of October 2017 did not affect the frequency of their visits to these areas (Table 2).

**Table 2:** Clusters’ profiles regarding the knowledge about the *Matas do Litoral,* visiting frequency and motivations. Values in bold correspond to the highest values when statistically significant differences exist. a) The assumptions of Chi-square test were not observed. *Only the values corresponding to ‘yes’ are presented

Knowledge about the *Matas do Litoral*, visiting frequency and motivations	Total sample (*N* = 1000)		Clusters			Chi-square test	
	*N*	%	Cluster 1	Cluster 2	Cluster 3	*χ*^2^	*p* value
			*The moderately interested* (*N* = 240, 24%)	*The comprehensively interested* (*N* = 350, 35%)	*The environmentally interested* (*N* = 410, 41%)		
			***%***	***%***	***%***		
Knows the Matas do Litoral’*							
MN Leiria	740	74.0	57.5	**80.6**	78.0	45.309	< 0.001
MN Urso	273	27.3	13.3	**44.3**	21.0	82.730	< 0.001
MN Pedrógão	600	60.0	51.1	**67.7**	58.5	16.701	< 0.001
MN Dunes of Quiaios	330	33.0	19.6	**43.4**	32.0	36.959	< 0.001
MN Dunes of Vagos	336	33.6	26.3	**39.7**	32.7	11.831	0.003
PF Dunes and Pinewoods of Mira	429	42.9	31.7	**55.7**	38.5	39.012	< 0.001
PF Dunes of Cantanhede	339	33.9	27.1	**42.6**	30.5	18.852	< 0.001
Visits the Matas do Litoral*							
MN Leiria	511	51.1	40.8	**58.6**	50.7	17.965	< 0.001
MN Urso	168	16.8	12.1	**26.0**	11.7	32.621	< 0.001
MN Pedrógão	301	30.1	25.0	30.6	31.7	4.304	0.1
MN Dunes of Quiaios	192	19.2	12.5	**23.4**	19.5	11.004	0.004
MN Dunes of Vagos	175	17.5	12.5	20.0	18.3	5.849	0.06
PF Dunes and Pinewoods of Mira	263	26.3	17.1	**34.3**	24.9	22.461	< 0.001
PF Dunes of Cantanhede	178	17.8	15.0	**22.0**	15.9	6.567	0.037
Motivations to visit the Matas do Litoral*							
Play Sports	98	9.8	7.1	**13.7**	8.0	9.493	0.009
Admire the landscape, contact with nature	351	35.1	22.9	**43.7**	34.9	27.049	< 0.001
Breathe fresh air	123	12.3	12.5	**16.6**	8.5	11.312	0.003
Hunt	12	1.2	2.5	1.1	0.5	a)	a)
Picnics	118	11.8	15.0	12.6	9.3	5.086	0.08
Events celebration	17	1.7	4.6	1.1	0.5	a)	a)
Pick pine needles and branches	20	2.0	2.5	3.1	0.7	a)	a)
Mushroom picking	10	1.0	2.5	1.1	0.0	a)	a)
Visit ancient buildings	5	0.5	0.8	0.9	0.0	a)	a)
Hiking and walking	260	26.0	25.0	21.1	**30.7**	9.187	0.010
Changes in the visits’ frequency after the wildfires) (*N* = 702)						32.529	< 0.001
Visits increased	30	4.2	**12.3**	3.1	1.4		
Visits decreased	313	44.0	33.3	**47.0**	46.2		
Visits remained the same	368	51.8	**54.3**	49.8	52.4		

Unsurprisingly, the mega-fire events are perceived very negatively, “as catastrophes, tragedies, and calamities” (45.6%), caused by “incompetence, negligence, and abandonment” (35.5%), resulting in feelings of “sadness and pain” (32.2%). 47% of the respondents generally blame the “State/central government*”* for these events (Table 3). Despite their significant impacts, only 9.7% of the respondents were directly affected by the mega-fires, while 33.4% knew someone who was. Among those who were directly affected, 83.5% did not receive any type of support (Table 3).

**Table 3:** Clusters’ profiles regarding the perceptions about the wildfires of October 2017 in the *Matas do Litoral* and responsibility for these events. *Only the values corresponding to ‘yes’ are presented. Values in bold correspond to the highest values when statistically significant differences exist

Perceptions about the wildfires of October 2017 in the *Matas do Litoral,* responsibility for these events and impacts*	Total sample (*N* = 1000)		Clusters			Chi-square test	
	*N*	%	Cluster 1	Cluster 2	Cluster 3	χ^2^	*p* value
			*The moderately interested* (*N* = 240, 24%)	*The comprehensively interested* (*N* = 350, 35%)	*The environmentally interested* (*N* = 410, 41%)		
			%	%	%		
Perceptions about the wildfires							
Crime	124	12.5	12.5	12.5	12.5	0.000	1.0
Catastrophe, tragedy, horror calamity	452	45.6	35.8	**53.8**	44.4	18.759	< 0.001
Sadness, pain	319	32.2	25.4	33.1	**35.3**	6.991	0.030
Incompetence, negligence, abandonment	352	35.5	**52.9**	26.5	32.8	45.357	< 0.001
Destruction, loss, devastation	141	14.2	12.5	9.6	**19.1**	14.648	< 0.001
Environmental causes	33	3.3	5.0	2.0	3.4	3.888	0.1
Non-intentional human causes	57	5.7	7.9	5.2	4.9	2.792	0.2
Revolt, indignation	68	6.9	5.8	9.0	5.6	3.846	0.1
Responsibility for the wildfires							
Forest Owners	77	8.5	**13.2**	6.8	7.1	8.412	0.015
Government/ State	424	47.0	**55.7**	32.6	53.7	39.384	< 0.001
Local government	181	20.0	22.4	14.8	**23.0**	8.018	0.018
Arsonists/ criminals	182	20.2	11.4	**28.7**	18.4	25.682	< 0.001
Loggers and other interested stakeholders	109	12.1	7.5	11.9	**15.1**	7.670	0.022
Climate change and other environmental aspects	115	12.7	9.6	14.8	12.9	3.195	0.2
Negligence, abandonment, lack of means and resources	101	11.2	**16.2**	9.4	9.6	7.818	0.020
ICNF—*Matas do Litoral* management body	76	8.4	7.0	9.4	8.5	0.936	0.6
Civil Protection-related entities	90	10.0	11.4	9.0	9.9	0.831	0.7
Citizens	152	16.8	18.4	16.5	16.2	0.559	0.8
Impacts experienced						22.217	< 0.001
Directly	97	9.7	5.4	**13.7**	8.8		
Not directly but knows someone affected	334	33.4	28.7	**37.7**	32.4		
No	569	56.9	**65.8**	48.6	58.8		
Support received						a)	a)
Government	6	6.2	0.0	8.3	5.6		
Personal donations	4	4.1	0.0	8.3	0.0		
Insurances	2	2.1	15.4	0.0	0.0		
Other	4	4.1	15.4	4.2	0.0		
None	81	83.5	69.2	79.2	94.4		
Will the *Matas do Litoral* recover from the wildfires?						37.781	< 0.001
Yes, they are already naturally regenerating	235	23.5	20.4	**27.4**	22.0		
Yes, they are already being recovered by ICNF	150	15.0	**18.3**	13.7	14.1		
No, nothing is being done	140	14.0	**20.4**	8.9	14.6		
No, the destruction is irreversible	63	6.3	**9.2**	6.3	4.6		
Maybe, but recovery will take a long time	412	41.2	31.7	43.7	**44.6**		

When asked about the most important dimensions for the adequate management of the *Matas do Litoral,* environmental-related aspects, such as “promoting the cleaning of the forests” (4.72), “promoting animal and plant diversity” (4.68), and “protecting and preserving the *Matas*” (especially from wildfires) (4.67), stand out. Inversely, economic dimensions, like “ensuring wood production and financial income” (3.97) and the “privatization of forests” (3.22) are much less valued (Table 4). Despite these clear priorities regarding management, only 21.4% of respondents declare to know the management entity of the *Matas* while just 24.5% are willing to be involved in the management decisions (Table S3).

**Table 4:** Clusters’ Profiles regarding the most important dimensions for an adequate management of the *Matas do Litoral*. *Measured using a Likert-type scale from 1—‘Not important’ to 5—‘Extremely important’. Values in bold correspond to the highest values when statistically significant differences exist

Most important dimensions for an adequate management of the Matas do Litoral*	Total sample (*N* = 1000)	Clusters			ANOVA	
	Mean	Cluster 1	Cluster 2	Cluster 3	*F* value	*p* value
		*The moderately interested* (*N* = 240, 24%)	*The comprehensively interested* (*N* = 350, 35%)	*The environmentally interested* (*N* = 410, 41%)		
		Mean	Mean	Mean		
Use technical and scientific knowledge	4.46	3.84	**4.80**	4.53	152.267	< 0.001
Make decisions on forest stands	4.44	3.89	**4.83**	4.44	134.775	< 0.001
Protect and preserve the woods (e.g., from wildfires)	4.67	4.12	**4.92**	4.78	199.035	< 0.001
Have human and financial resources to promote forest surveillance	4.59	4.05	**4.86**	4.67	157.741	< 0.001
Preserve the forest and the historical patrimony for the future generations (e.g., ancient trees, ancient buildings)	4.60	3.96	**4.89**	4.73	240.075	< 0.001
Promote leisure and recreation in the forests	4.40	3.76	**4.81**	4.43	172.423	< 0.001
Promote other economic forests uses (e.g., agriculture, grazing)	4.29	3.80	**4.75**	4.17	99.860	< 0.001
Ensure wood production and financial income	3.97	3.73	**4.67**	3.50	133,739	< 0.001
Reinvest the income from the wood sale into the forests	4.27	3.86	**4.78**	4.07	89.365	< 0.001
Ensure the production of non-wood products and financial income (e.g., biomass, resin)	4.26	3.83	**4.79**	4.04	125.378	< 0.001
Promote the forest cleaning	4.72	4.18	**4.94**	4.85	199.276	< 0.001
Promote the diversity of animals and plants	4.68	4.04	**4.91**	4.86	269.525	< 0.001
Ensure afforestation with *pinus pinaster*	4.45	3.91	**4.76**	4.50	97.346	< 0.001
Privatize the forests	3.22	3.26	**3.60**	2.88	25.387	< 0.001
Involve municipalities and parishes in management decisions	4.45	3.89	**4.75**	4.51	109.113	< 0.001
Involve local population and stakeholders in management decisions	4.42	3.74	**4.80**	4.50	175.465	< 0.001

In considering the future of these public forests, 41.2% of the respondents believe they "might recover, but it will take a long time” (Table 3). 37.3% want the *Matas* will be “well-managed and planned” in the future, with 37.2% desiring their continuity as “spaces for protecting nature and biodiversity” (Table S4).

### Clusters profiles

Statistically significant differences were found among the three clusters identified regarding the relevance attributed to each of the specified uses and functions of the *Matas do Litoral* and the importance attributed to these forests at various territorial scales (Table 1); their perceptions of the *Matas* prior to the mega-fires (Table S2); knowledge and familiarity with these areas and visiting habits and motivations (Table 2); perceptions of the mega-fires, responsibility for these events, impacts experienced and support received (Table 3); views on the most important features for an adequate forest management (Table 4); willingness to participate in management-related decisions (Table S3), perceptions of the future of the *Matas do Litoral* (Table S4); and respondents’ sociodemographic profiles (Table S1).

Cluster 1—“The Moderately Interested”—(*N* = 240, 24%) include the respondents that generally assign scores below 4.0 to almost all the functions and uses of the *Matas do Litoral*. The few exceptions regard “the preservation of nature and landscapes” (4.12), “improving air quality” (4.10), and “preserving the diversity of animals and plants” (4.00). However, even these scores are considerably lower than those given within the other two clusters. “The Moderately Interested” tend to perceive the *Matas do Litoral*, prior to the October 2017 mega-fires, as dangerous and insecure places, prone to wildfires, and are more likely to associate the *Matas* with specific forest areas and localities. Respondents within this cluster tend to attribute lower relevance to the *Matas* at all the territorial scales (parish, municipality, region, country and internationally). They also have less tendency to know and visit these forests, which results in less engagement in related leisure and recreation activities.

Following the mega-fires of October 2017, “The Moderately Interested” maintained the frequency of their visits to these forests, with a few even reporting an increase. Respondents in this cluster attribute those mega-fires primarily to “incompetence, negligence, and abandonment,” mainly blaming the “State/central government,” the “negligence, abandonment, and lack of means and resources,” and “forest owners.” “The Moderately Interested” are more likely to believe the *Matas do Litoral* will not recover from the fires’ impacts, citing either a lack of action or the irreversibility of the damages. However, some respondents in this group do feel that the ICNF is already making some efforts to recover the burned areas. Unsurprisingly, this cluster assigns lower relevance to (almost) all the forest management dimensions considered, with most scores falling below 4.00.

Respondents in this cluster are more likely to not knowing the management entity of the *Matas do Litoral*, showing less interest in participating in the related decision-making processes. This is due both to “lack of interest” (the main reason) and “lack of time.” Regarding the future, “The Moderately Interested” are more likely to view these forests as “spaces of wealth” and devoted to “the protection of heritage.” This group primarily includes individuals who were not directly affected by the wildfires, aged 18–24 years, and 25–64 years, with secondary or university degrees. Compared to the second cluster,, this group includes fewer forest owners.

Cluster 2 (*N* = 350, 35%)—“The Comprehensively Interested”—comprises the respondents who are more likely to assign high ratings (close to 5.0) to all the functions and uses of the *Matas do Litoral*, therefore perceiving these forests’ economic, environmental and conservation values as very important, demonstrating a multifunctionality-oriented view. They are more likely to portray the *Matas* (before the wildfires of 2017) as vital “sources of fresh air and oxygen” and essential for “life and health.” They tend to consider these forests as extremely important at all territorial levels, especially at the regional and national scales. This group typically includes respondents who are well-acquainted with the *Matas do Litoral*, frequently visiting them mainly for “admiring the landscape and connecting with nature” and “breathe fresh air.” However, in consequence of the mega-fires, “The Comprehensively Interested” have decreased the frequency of their visits to the *Matas*. They perceive those events as “catastrophes, tragedies, horror, or calamities,” primarily blaming the “arsonists/ criminals.”

Respondents in this cluster were more likely to have been directly affected by these events, and to know someone who has been. Among those directly impacted, “private donations” and “government assistance” were the most common types of support received. Respondents in this cluster believe that, following the mega-fires, the *Matas do Litoral* are already naturally regenerating. With the exception of the “privatization of the *Matas*” (scored at 3.60), this group consistently scores all the other management-related dimensions higher (above 4.67) than the other two clusters. “The Comprehensively Interested” are more likely to know which entity manages the *Matas do Litoral* and are eager to participate in the management-related decisions. However, compared to the other clusters, they offer a wider variety of reasons for not participating, especially the lack of knowledge and information about the participatory processes. Respondents in this cluster hope that the *Matas do Litoral* will become again “green” and “sources of fresh air” in the future. This cluster is more likely to include a higher number of forest owners, older individuals (over 65) with lower literacy levels (below secondary education).

Cluster 3—“The Environmentally Interested”—(*N* = 410, 41%) lies between the other two clusters in terms of the importance assigned to the various functions of the *Matas do Litoral*. They are more likely to prioritize functions related to environmental conservation while assigning less relevance to economic uses. Despite their pro-conservation views, their perception of the *Matas do Litoral,* prior to the mega-fires of 2017, tends to be negative, contrasting with the views of the respondents from the other two clusters. “The Environmentally Interested” are more likely to describe these forests as “abandoned and neglected,” expressing feelings of “sadness and pain” due to a perceived “lack of adequate maintenance”*.*

Although less than “The Comprehensively Interested,” this group is also more likely to consider the *Matas* as important at all the territorial scales. While they are more familiar than “The Moderately Interested” with the *Matas do Litoral*, their knowledge and visiting habits are still significantly lower than those of “The Comprehensively Interested.” “Hiking and walking” activities are the primary motivation of their visits to the *Matas*. Like “The Comprehensively Interested,” they have decreased the frequency of their visits after the mega-fires. Being the second group of respondents most directly affected by these fires, they frequently express feelings of “sadness and pain” and “loss and devastation” regarding these events, mainly blaming “local government” representatives and “loggers and other interested stakeholders.”

Respondents within this cluster are more likely to foresee the recovery of the *Matas*, although it may take a long time. “The Environmentally Interested” are much less inclined than the other two clusters to view the “privatization of the forests” and “ensuring wood production and financial income” as important dimensions for effective management of these areas. Compared to “The Comprehensively Interested,” respondents in this group are less likely to know the *Matas do Litoral* managing entity*,* as well as less interested in participating in the decision-making processes, primarily because they feel their opinions are not considered. They are more likely to wish that the *Matas* return to being places for “nature and biodiversity conservation” in the future. This group includes the lowest number of forest owners. Respondents in this cluster are typically aged between 25 and 64 years and hold university degrees.

## Discussion

The main aim of this research was to analyze the contrasting social perceptions of public forests’ functions and values, following mega-fire events. To achieve this aim, the study focused on the *Matas do Litoral,* Portuguese public-managed forests that were severely impacted by the mega-fires of October 2017. The study is based on the results of a survey conducted to a sample of 1000 residents in the adjacent parishes. Three distinct clusters emerged from the hierarchical cluster analysis performed—“The Moderately Interested,” “The Comprehensively Interested” and “The Environmentally Interested”—according to their perceptions regarding the *Matas do Litoral* functions.

The empirical evidence discussed here corroborates earlier findings by, among other, Rametsteiner and Kraxner (2003), Carvalho-Ribeiro and Lovett (2011), Edwards et al. (2012), and Valente et al. (2015) regarding the differences between Central and Southern European citizens’ perceptions about forest preferences and functions. Although these studies reveal minimal significant differences between the two contexts, they also stress that Southern European citizens tend to attribute greater relevance to functions related to biodiversity conservation and protection against natural hazards, namely wildfires (e.g., Rametsteiner and Kraxner 2003; Valente et al. 2015; Ranacher et al. 2017).

As mega-fires directly impact the functions and services that forests may provide (as noted by Frick et al. 2018 and Newman Thacker et al. 2025), their contribution to shape social perceptions about forests is significant, as the case analyzed here demonstrates. This is evident in the responses of the two larger clusters studied, whose activities and experiences related to the *Matas do Litoral* were significantly affected by the mega-fires of October 2017. They describe these events in a very negative and emotional manner (in line with the conclusions of the systematic literature review performed by Newman Thacker et al. 2025). On the contrary, the smaller cluster that presents an overall lower interest and weaker connections to the *Matas*, perceives those events in a less emotional fashion, objectively attributing them to the incompetence and negligence of the Portuguese government.

These findings clearly demonstrate that stronger connections to forests lead to perceptions deeply shaped by emotions, in line with the findings of Lidskog et al. (2019). This suggests that individuals’ perceptions of wildfires and their responsibility are significantly influenced by personal experiences. In turn, the direct experience of these impacts is crucial in shaping social perceptions of forests functions and values. These results also highlight the significant role played by knowledge and familiarity with forests in the respondents’ perceptions, resulting in diverse viewpoints among the three clusters analyzed, in line with the conclusions of previous studies (e.g., Rametsteiner et al. 2009; Fabra-Crespo et al. 2012; Paletto et al. 2013b; Ciesielski and Sterenczak 2018; Frick et al. 2018; Valente et al. 2024).

Therefore, the stronger connection with the *Matas do Litoral*, alongside respondents’ frequent visits and engagement in activities—especially demonstrated by “The Comprehensively Interested”—evinces a greater familiarity and knowledge with these forests that definitively contribute to shape these individuals’ perceptions and higher interest (as also noted by Frick et al. 2018). Conversely, lower visitation habits and reduced engagement in recreational (and other) activities are often associated with lower familiarity and knowledge levels, as well as with an overall lack of concern and interest in forests, as demonstrated by “The Moderately Interested.” As a result, the three clusters analyzed here demonstrate varying levels of interest and concern regarding the *Matas do Litoral*. These are influenced by respondents’ various degrees of familiarity and knowledge, which in turn also influence their assessments of the *Matas* functions and values.

“The Comprehensively Interested” are also more likely to hold a multifunctional perception of forests and the services they can provide, as noted, among other, by the studies of Nijnik et al. (2010) and Pastorella et al. (2016). The higher value placed by the larger cluster—“The Environmentally Interested”—in nature and biodiversity functions, in turn, reinforces the conclusions of Rametsteiner and Kraxner (2003), Rametsteiner et al. (2009) and Valente et al. (2015) regarding the greater relevance attributed to these specific functions by Southern European citizens. At the same time, these findings corroborate the conclusions of Paletto et al. (2013b) regarding society’s shift from instrumental-oriented values to more intrinsic-related ones clearly recognizing the relevance of forest ecosystems and inherent environmental values over the more traditional (economic and productive) functions. However, as our findings also demonstrate through the sometimes overlapped and not clear-cut perceptions held by the three clusters analyzed, both types of values seem to coexist, also being influenced by individuals’ socioeconomic characteristics.

The relevance of socioeconomic features in shaping social preferences and perceptions of forests has been profusely studied over recent years (e.g., Schmithüsen and Seeland 2006; Edwards et al. 2012; Paletto et al. 2013b; Valente et al. 2015; Barona et al. 2022). Our findings specifically demonstrate the relevance of age, literacy level, forest ownership and economic dependency on forests in determining the different perceptions held by the three clusters, while contrary to the abovementioned studies, gender is not so relevant. Forest ownership and economic dependency help to explain the multifunctional perception held by “The Comprehensively Interested,” together with their higher levels of familiarity and knowledge about the *Matas do Litoral*. The greater focus on environment conservation-related functions held by “The Environmentally Interested” seems to be, in turn, mainly influenced by their higher literacy levels, while the lower interest and concern of “The Moderately Interested” may be explained by their younger age, lower familiarity and economic dependency on forestry.

All these aspects are also relevant in shaping social perceptions regarding forest management issues, as the differences in the three clusters analyzed here reveal. Consequently, while more aware and concerned respondents tend to attribute higher scores to all the management dimensions considered in this study and to express their willingness to engage in the related decision-making processes, the less concerned individuals reveal a general lack of interest in almost all the management practices, along with a lower willingness to participate in the related decisions. In the same vein, forest ownership and economic dependency are important aspects supporting the relevance given to both economic and environmental-oriented management dimensions. Particularly, the emphasis on the preservation of biodiversity and environmental protection-oriented management practices, aligns with the conclusions of Ramesteiner et al. (2009) and Valente et al. (2015) regarding Southern European citizens growing concerns. These are critical aspects to be considered by decision-makers and managers, the State agency responsible for the management of the *Matas do Litoral* included.

As discussed above, sustainable forest management has evolved from focusing on forests’ productive functions to the progressive inclusion of a broader range of services and values to balance sociocultural, economic and ecological concerns and needs of both the current and the future generations. This change also supports the integration of the diverse stakeholders in the management processes and the understanding of their multiple interests, concerns and needs. Yet, despite the willingness to participate in the management-related decisions, demonstrated by an important part of the respondents analyzed here, a higher level of participation and involvement seems to be compromised by the lack of information alongside the perception that citizens’ opinions are not adequately considered in those processes. Specifically, and considering that the participation models followed in Portugal (and, as stressed by Fernandes et al. 2024, in other Southern European countries) are still centralized and based on meager (and often scarce) information and consultation strategies, our findings clearly suggest the need to develop communication channels and practices, and to create alternative models of citizen involvement. These are again critical issues to be considered by forests’ management entities in general, and within the *Matas do Litoral* in particular, if more involvement and engagement of stakeholders is intended, aiming at sustainable and inclusive forest management strategies and practices.

Despite the substantial challenges that persist, various tools and strategies for engaging communities and stakeholders can serve as valuable references, as demonstrated by Fernandes et al. (2024), Figueiredo et al. (2024a, b) and Ottolini et al. (2023). Furthermore, although significant progress is still to be made in establishing sustainable and participatory management practices in Portugal, the aftermath of the 2017 mega-fires marked a pivotal turning point. Notably, the creation of the Agency for the Integrated Management of Rural Fires (AGIF) in 2018—a public institute mandated to catalyze the shift from reactive emergency responses to integrated, prevention-focused fire governance—represented an important step to improve institutional coordination and promote the involvement of local communities as essential partners in fire risk mitigation.

Despite the theoretical and practical contributions of this study, our results are not without limitations. The focus on public-managed forests hampers the possibility to contribute to a more comprehensive understanding of social perceptions regarding the functions and values of forests in Portugal, a country in which only 3% of the forest area is publicly managed. Furthermore, while surveys present advantages in terms of speed and efficiency regarding data collection and analysis, particularly with large populations, they generally lack the flexibility found in qualitative methods like interviews and focus groups. Therefore, using qualitative tools alongside questionnaires, in future research, can reveal the respondents’ narratives and provide a more detailed assessment of their perceptions.

Notwithstanding, these limitations do not overshadow the pioneering effort undertaken by this study in examining the social perceptions about forests’ functions and values in a Southern European country, a context generally overlooked by scientific research. Despite the similarities with other studies conducted elsewhere, our findings highlight important differences related to contextual sociocultural and economic specificities. In this vein, conducting similar studies in both analogous and diverse contexts would be a valuable research avenue, especially to better understand the role played by cultural and social features in shaping social perceptions of forests. Furthermore, such comparative studies could significantly contribute to designing and implementing more informed sustainable forest management decisions and strategies at both EU and national levels, able to effectively include the diversity of stakeholders, perceptions, needs, and interests, at the same time promoting rewarding citizens involvement in management decision-making processes.

## Conclusion

This study offers a novel contribution to the understanding of social perceptions surrounding publicly managed forests in a Southern European context, where such perspectives have remained largely underexplored. By investigating the values, meanings, and expectations that individuals associate with forest areas, our findings emphasize the critical societal relevance of forests.

The analysis demonstrates that social perceptions are significantly shaped by factors such as socioeconomic characteristics, forest ownership, economic dependence on forestry, and levels of knowledge and familiarity with forest environments. Notably, stronger knowledge and connections with forests correlate with greater concern, heightened recognition of forest functions and services, and increased interest in participating in forest governance. These insights underline the importance of integrating social perceptions into forest policy and management decisions. To promote more sustainable and inclusive forest management—especially within public-managed areas and regions where participatory practices remain limited—forest management entities should invest in improving and decentralizing communication mechanisms and strengthening participatory strategies.

## Supplementary Information

Below is the link to the electronic supplementary material.

Supplementary file1 (PDF 449 kb)

## Data Availability

Data will be made available on reasonable request.

## Footnotes

1 https://geocatalogo.icnf.pt/geovisualizador/refloa/.
