# Supplementary material for: What are forests for? Social perceptions of the functions of public-managed forests following mega-fire events
Source: Ambio. 2025 Jun 3;54(11):1939–53. doi: 10.1007/s13280-025-02200-1 (PMC12480173; doi:10.1007/s13280-025-02200-1)
Supplement: Supplementary file 1 — Supplementary file1 (PDF 449 kb) [file 13280_2025_2200_MOESM1_ESM.pdf]

AMBIO

Supplementary Information

This supplementary information has not been peer reviewed.

Title: **Sociodemographic profiles of the respondentes;**

Clusters' profiles regarding:

- **The perceptions of the 'Matas do Litoral' before the October 2017 wildfires;**
- **The interest in participating in the 'Matas do Litoral' management-related decisions and knowledge on the management entity;**
- **The perceptions about the future of the 'Matas do Litoral'**

Table S1 – Sociodemographic profiles of the respondents

Sociodemographic Profile	Total sample (N=1000) N	%	Clusters: Cluster 1 *The Moderately Interested* (N=240, 24%) %	Cluster 2 *The Comprehensively Interested* (N=350, 35%) %	Cluster 3 *The Environmentally Interested* (N=410, 41%) %	Chi-square Test: χ2	*p*-value
**Gender**						1.713	0.4
Female	517	51.7	51.7	49.1	53.9		
Male	483	48.3	48.3	50.9	46.1		
**Age**						10.691	0.003
18-24 years old	104	10.4	**11.7**	11.1	9.0		
25-64 years old	617	61.7	**65.8**	55.7	64.4		
65 or more years	279	27.9	22.5	**33.1**	26.6		
**Municipality of Residence**						17.505	0.1
Cantanhede	44	4.4	2.1	6.3	4.1		
Figueira da Foz	92	9.2	6.7	10.9	9.3		
Leiria	21	2.1	1.3	2.0	2.7		
Marinha Grande	440	44.0	50.4	41.4	42.4		
Mira	136	13.6	10.0	14.3	15.1		
Pombal	112	11.2	12.5	10.0	11.5		
Vagos	155	15.5	17.1	15.1	14.9		
**Marital Status**						6.128	0.4
Single	275	27.5	30.8	25.7	27.1		
Married/cohabitating	606	60.6	57.5	62.3	61.0		
Divorced/ separated	75	7.5	9.2	6.6	7.3		
Widowed	44	4.4	2.5	5.4	4.6		
**Level of Education**						14.246	0.007
Up to 3rd cycle	250	25.0	16.7	**30.3**	25.4		
Secondary education	311	31.1	**33.8**	29.1	31.2		
University education	439	43.9	**49.6**	40.6	43.4		
**Net monthly household income**						a)	a)
705 € and below	89	8.9	5.4	10.0	10.0		
706 € to 1500 €	315	31.5	35.0	33.4	27.8		
1501 € to 3000 €	357	35.7	35.4	35.7	35.9		
3001 € to 4500 €	68	6.8	8.8	4.6	7.6		
More than 4501 €	15	1.5	1.7	1.7	1.2		
**Forest ownership ***	214	21.4	25.0	**26.9**	14.6	19.204	<0.001
**Forest properties border with ML***	46	21.5	21.7	23.4	18.3	0.560	0.8

*Only the values corresponding to 'yes' are presented

a) The assumptions of Chi-square test were not observed

Note: Values in bold correspond to the highest values when statistically significant differences exist

Table S2 - Clusters' profiles regarding the perceptions of the *Matas do Litoral* before the October 2017 wildfires

**Perceptions of *Matas do Litoral* ***	**Total sample (N= 1000)**		**Clusters**			**Chi-square Test**	
	**N**	**%**	**Cluster 1 *The Moderately Interested* (N=240, 24%) %**	**Cluster 2 *The Comprehensively Interested* (N=350, 35%) %**	**Cluster 3 *The Environmentally Interested* (N=410, 41%) %**	**$\chi 2$**	***p*-value**
Forests, Pinewoods, Trees	105	10.7	11.5	11.6	9.6	0.990	0.6
Fresh air, Oxygen	189	19.3	14.9	**27.5**	15.2	21.543	<0.001
Beauty	113	11.6	9.4	12.2	12.3	1.470	0.4
Green, Nature, Environment	295	30.2	20.4	30.4	30.5	0.102	0.9
Abandonment, Negligence, Lack of Maintenance	202	20.7	23.8	13.4	**24.8**	16.394	<0.001
Life, Essential, Health	89	9.1	3.8	**13.7**	8.4	16.835	<0.001
Peace, Tranquility, Freedom, Leisure	89	9.1	8.5	7.5	10.8	2.622	0.3
Protection, Conservation, Planning	142	14.5	16.2	12.2	15.5	2.220	0.3
Sadness, Pain	35	3.6	0.9	3.9	**4.9**	7.251	0.027
Wildfires, Danger, Insecurity	94	9.6	**15.3**	8.1	7.6	11.594	0.003
Patrimony, Memory	33	3.4	3.4	3.9	2.9	0.490	0.7
Specific Forests and Localities	109	11.2	**13.6**	6.9	13.3	9.448	0.009

*Only the values corresponding to 'yes' are presented

Note: Values in bold correspond to the highest values when statistically significant differences exist

Table S3 – Clusters' Profiles regarding the interest in participating in the *Matas do Litoral* management-related decisions and knowledge on the management entity

**Interest in participating in the Matas do Litoral management-related decisions and knowledge of the managing entity**	**Total sample (N=1000)**		**Clusters**			**Chi-square Test**	
	**N**	**%**	**Cluster 1** ***The Moderately Interested*** **(N=240, 24%) %**	**Cluster 2** ***The Comprehensively Interested*** **(N=350, 35%) %**	**Cluster 3** ***The Environmentally Interested*** **(N=410, 41%) %**	**$\chi 2$**	***p*-value**
**Knowledge on the management entity***	214	21.4	13.3	**28.3**	20.2	19.476	<0.001
**Would like to be involved in management decisions***	245	24.5	17.1	**32.6**	22.0	20.904	<0.001
**Reason for not being involved**						21.512	0.001
Lack of interest	98	28.5	**37.4**	21.7	28.6		
Lack of time	63	18.3	**25.3**	16.7	15.0		
Feels like the opinion is not considered	125	36.3	31.9	35.8	**39.8**		
Other reason	58	16.9	5.5	**25.8**	16.5		

*Only the values corresponding to 'yes' are presented

Note: Values in bold correspond to the highest values when statistically significant differences exist

Table S4 – Clusters' Profiles regarding the perceptions about the future of the *Matas do Litoral*

**Perceptions on the *Matas do Litoral* future***	**Total sample (N=1000)**		**Clusters**			**Chi-square Test**	
	**N**	**%**	**Cluster 1 *The Moderately Interested* (N=240, 24%) %**	**Cluster 2 *The Comprehensively Interested* (N=350, 35%) %**	**Cluster 3 *The Environmentally Interested* (N=410, 41%) %**	**χ2**	***p*-value**
Green	216	21.8	12.5	**32.5**	18.3	30.013	<0.001
Spaces for the protection of nature and biodiversity	368	37.2	42.9	25.2	**44.1**	32.695	<0.001
Well managed and planned	369	37.3	32.5	38.8	38.9	3.135	0.2
Spaces for leisure	152	15.4	18.8	15.9	12.9	4.134	0.1
Spaces to breathe fresh air	140	14.2	6.7	**20.9**	12.9	24.423	<0.001
Spaces for the protection of patrimony	62	6.3	**8.8**	3.8	6.9	6.487	0.039
Beautiful, alive, joyful	119	12.0	12.5	14.5	9.7	4.183	0.1
Reforested, recovered	60	6.1	6.3	4.1	7.7	4.287	0.1
Spaces of wealth	66	6.7	**13.3**	4.9	4.2	22.724	<0.001
Surveyed, safe	88	8.9	11.7	7.5	8.4	3.175	0.2

*Only the values corresponding to 'yes' are presented

Note: Values in bold correspond to the highest values when statistically significant differences exist
